# What do we know about communicating risk? A brief review and suggestion for contextualising serious, but rare, risk, and the example of cox-2 selective and non-selective NSAIDs

**DOI:** 10.1186/ar2373

**Published:** 2008-02-07

**Authors:** R Andrew Moore, Sheena Derry, Henry J McQuay, John Paling

**Affiliations:** 1 Pain Research and Nuffield Department of Anaesthetics, University of Oxford, Oxford Radcliffe NHS Trust, The Churchill, Headington, Oxford OX3 7LJ, UK; 2 Risk Communication Institute, 5822 NW 91st Boulevard, Gainesville, Florida 32653, USA

## Abstract

**Background:**

Communicating risk is difficult. Although different methods have been proposed – using numbers, words, pictures or combinations – none has been extensively tested. We used electronic and bibliographic searches to review evidence concerning risk perception and presentation. People tend to underestimate common risk and overestimate rare risk; they respond to risks primarily on the basis of emotion rather than facts, seem to be risk averse when faced with medical interventions, and want information on even the rarest of adverse events.

**Methods:**

We identified observational studies (primarily in the form of meta-analyses) with information on individual non-steroidal anti-inflammatory drug (NSAID) or selective cyclooxygenase-2 inhibitor (coxib) use and relative risk of gastrointestinal bleed or cardiovascular event, the background rate of events in the absence of NSAID or coxib, and the likelihood of death from an event. Using this information we present the outcome of additional risk of death from gastrointestinal bleed and cardiovascular event for individual NSAIDs and coxibs alongside information about death from other causes in a series of perspective scales.

**Results:**

The literature on communicating risk to patients is limited. There are problems with literacy, numeracy and the human tendency to overestimate rare risk and underestimate common risk. There is inconsistency in how people translate between numbers and words. We present a method of communicating information about serious risks using the common outcome of death, using pictures, numbers and words, and contextualising the information. The use of this method for gastrointestinal and cardiovascular harm with NSAIDs and coxibs shows differences between individual NSAIDs and coxibs.

**Conclusion:**

Although contextualised risk information can be provided on two possible adverse events, many other possible adverse events with potential serious consequences were omitted. Patients and professionals want much information about risks of medical interventions but we do not know how best to meet expectations. The impact of contextualised information remains to be tested.

## Introduction

Many factors contribute to an incomplete understanding and evidence base for risk and risk presentation. We should not be surprised when both patients and professionals are confused about risk, about competing risks, and about comparing risks with benefits. Decisions are based on facts and emotions, both of which may be manipulated, and it may well be that emotions dominate the facts. This is important in the framework of medical decision-making and specifically in the choice of pharmacological and interventional therapies for individuals.

Risk has two main components. One is that of chance, the pure statistical likelihood that an event will happen (probability). The other is that of a bad outcome – danger, injury, harm or loss – together with an indication of severity. To some extent the term is used commonly to process or communicate the product of probability and severity, and the complexities have been reviewed elsewhere [1].

We can recognise three main areas that have to be considered to help professionals understand their patients' risk, and patients to understand their own risk. Broadly these can be aggregated under the headings of perception (influences on how individuals and populations relate to risk information), presentation (how information – data – can be conveyed, and possibly manipulated, for clarity or impact), and pertinent facts (accurate data with clear, decisive relevance to the matter in hand, and which may be used as the basis of future outcomes). These broad areas are not independent of each other, but it helps understanding to try to organise the many different facets of risk.

'Everything is poison, there is poison in everything. Only the dose makes a thing not a poison.' Paracelsus might have been intrigued by the controversy that has arisen over the cardiovascular adverse effects that have lately been associated with traditional NSAIDs and selective cyclooxygenase-2 inhibitors (coxibs) [2]. Traditional NSAIDs have long been associated with upper gastrointestinal bleeding, renal impairment, and congestive heart failure, and, more recently, with injury to the lower bowel. The only expected benefit of coxibs over NSAIDs was reduced levels of upper gastrointestinal bleeding.

NSAIDs and coxibs have become some of the most studied drugs ever, with at least 145,000 patients enrolled in randomised trials [3], and with up to 3.5 million patients in observational studies [4]. There is unprecedented information on different adverse events associated with particular drugs, especially for the outcomes of upper gastrointestinal bleeding and cardiovascular risk.

Different drugs, even within a class, can have different rates of particular adverse events. For NSAIDs there are large differences between drugs and between different doses of the same drug in terms of upper gastrointestinal bleeding. Individual patient meta-analysis showed that low-dose ibuprofen was not different from non-use, whereas high-dose naproxen had an odds ratio of 16 [5]. In observational and other studies of NSAIDs there were large differences between drugs [6]. Similarly, differences between individual coxibs are apparent for gastrointestinal bleeding [7], and between individual coxibs and NSAIDs for myocardial infarction [4,3,8].

This review set out to do three things: to examine the background to our understanding and perception of risk; to examine how risk can be presented, and explore the possibility of using a common outcome, death, and contextualising information on non-medical life risks with a presentation involving numbers, words, and pictures, based on visual aids introduced by Paling [9]; and to explore how competing risks of death from gastrointestinal bleeding or cardiovascular events with NSAIDs and coxibs might be presented by using this method.

The only certainty is that there is uncertainty. We wish to emphasise that these explorations are not intended to be definitive; indeed, they cannot be without extensive testing. However, given the growing emphasis of patient involvement in decision-making, methods have to be developed that can deliver risk information effectively.

## Materials and methods

We initially searched PubMed using a number of free-text terms for the particular area of interest. Thus for literacy, for instance, we sought articles with literacy in the title. Other searches were aimed at numeracy, risk, and risk presentation or perception. An iterative search process was then applied to identify additional studies; this involved checking the 'Web of Knowledge Cited References', and the 'Related Articles' link in PubMed using details of retrieved studies from the initial search. When the iterative process indicated alternative search terms, we repeated searches using these new terms. Terms were generally restricted to title only, at least initially, to avoid impossibly large numbers of references using words with many other common meanings (such as relative risk). We also checked the bibliographies of any relevant studies, risk websites (see [10], for instance) and books, reviews and articles on risk presentation. We looked for full journal-published articles without language restrictions.

## Results

### Background to risk perception

#### Literacy and numeracy

An inability to handle words or numbers at an appropriate level (literacy and numeracy skills) are fundamental to communicating risk probability or severity. Illiteracy in patients is known to be a barrier to communication. In a survey of 127 rheumatology patients in Glasgow [11], 3 were unable to read and 18 were functionally illiterate, so that 17% (1 in 6) would at best struggle with patient education material and 1 in 20 could not read prescription labels. An identical value of 17% with limited reading ability was found in 999 diabetic patients in primary care in Vermont [12].

Health numeracy has been provided with a set of definitions [13]. Using three simple questions to test for numeracy, Sheridan [14,15] showed that 5% (1 in 20) of US medical students and 71% (7 in 10) of patients at an internal medicine clinic could answer only one or none correctly. Half (1 in 2) of patients attending an anticoagulation clinic in North Carolina had numeracy and literacy skills that would limit their understanding [16].

#### Risk information that people want

A large study of 3,500 adults in Kansas indicated that 90% of them wanted information on all adverse events (not just serious adverse events) occurring in at least one person in every 100,000 [17]. This standard, if real, poses challenges in obtaining and communicating information on risk.

#### How the general public responds to risk information

People consistently overestimate rare risk and underestimate common risk. This was first shown for estimates of mortality three decades ago [18], and has been confirmed more recently [19] to demonstrate that the trend is common throughout society, although more educated and perhaps older people with more life experience have more accurate risk beliefs.

Where causes of death involved fewer than 10 deaths a year in the USA (fireworks, measles, botulism), overestimation was by almost two orders of magnitude [19]. Where causes of death involved many deaths a year (100,000 to 700,000 deaths: stroke, cancers, heart disease), underestimation was almost one order of magnitude. At the extremes, then, people overestimate rare risks by 100-fold or more, whereas they underestimate common risks by a factor of 10. The degree of overestimation or underestimation is startling.

Interestingly, both studies [18,19] showed that people were likely to judge the level of risk correctly when the risk was associated with about 1,000 deaths per year in the USA. It is also worth noting that different societies can have very different perceptions of the same risk. An important determinant may well be the state of technological development [20]. How this societal attitude relates to or affects individual attitude is not understood.

Attitudes to risk, at least to drug therapy, can be affected by direct-to-consumer advertising. Examining consumer responses to a US survey indicated that such advertising was associated with a greater willingness to talk with doctors about advertised drugs in those with a chronic condition, and that advertising made prescription drugs appear harmless [21]. US Food and Drug Administration research is quoted as showing that patients and physicians believe that consumer-directed advertising frequently overstates the benefits of drugs and understates the risks [22].

#### How patients respond to risk information

A number of small studies have assessed what patients think about risk and the effectiveness of interventions. There is a tendency for patients to overestimate the risk of something bad happening [23]. For instance, 65% (2 in 3) of women either overestimated or grossly overestimated their own chance of breast cancer [24]. Women also tended to overestimate the chance of harm with hormonal contraceptives and underestimate their effectiveness [25]. For other methods of contraception, women could overestimate effectiveness (female sterilisation or female condom) or underestimate it (hormonal implants and intrauterine devices).

In some circumstances, patients can be very risk averse, as a study of patients attending an emergency department in Boston demonstrated [26]. They were presented with a scenario in which they had come to hospital with chest pain that could not be diagnosed by standard procedures, and doctors asked them to participate in a trial using a safe and approved test involving a small amount of radioactivity that might help make a diagnosis. The study was about whether using the test in the emergency room rather than elsewhere in the hospital was acceptable, given that it had a very small level of risk. The trivial level of risk was presented in various ways, like being equivalent to 20 chest X-rays, smoking a small number of cigarettes, driving 150 miles, or breathing radon in a house for 2.5 years while living in Boston. Between 40% and 60% of patients would have refused to have the test in the emergency room, with more refusing than accepting it, however the risk was presented. Yet the additional risks were not only small, but equivalent to those they accepted as part of their life in any event, because they smoked, drove, or lived in Boston.

#### Dimensions of risk

**Figure 1:**
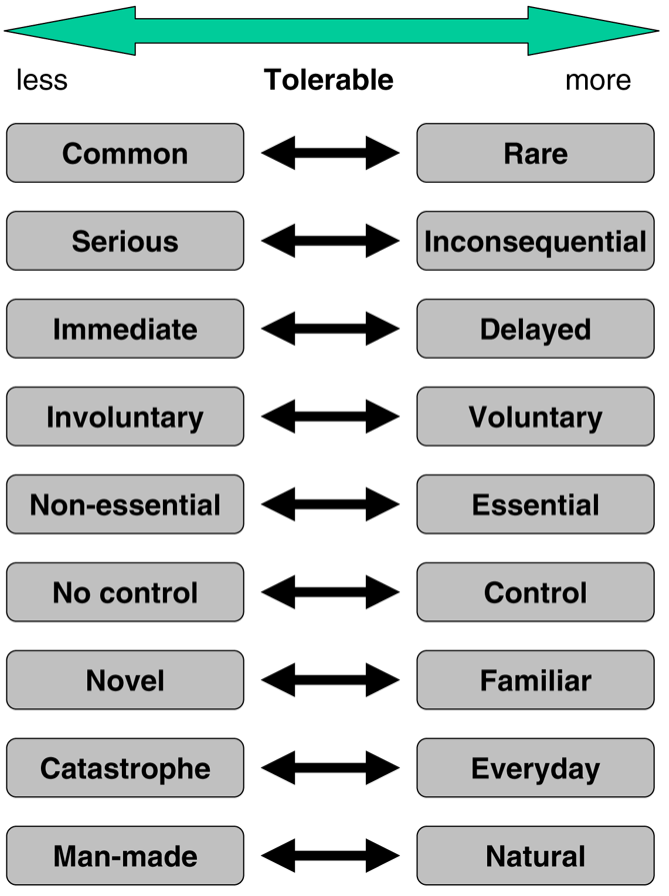
Some dimensions and qualities of risk and risk decisions.

Risk has a number of dimensions (Figure 1), with extremes that make a risk more or less tolerable. There is no good evidence about which dimensions are most important, how they affect patient or professional judgement, and in what circumstance they might do so.

It is generally assumed that risks over which individuals have no control are less acceptable than those over which they do have control, or that novel risks have greater impact than those with which we are familiar. Man-made risks appear to be worse than natural risks. For instance, the risks of radiation are often posed as a major concern, yet in the USA in 2002 there were no deaths from radiation, compared with 66 from lightning, 63 from cataclysmic storm, 31 from earthquake or other earth movements, and 9 from flood. There were 767 deaths of pedal cyclists in the USA in 2002 [27]. Some risks are not highly related to demographic variables such as sex or age (road traffic accidents, for example). Others, such as the risk of death by choking, are so related; here annual risk is lowest at 1 in 1,000,000 in children aged 5 to 18 years, but approaches 1 in 1,000 in the over-90s.

These are trivial compared with the top two causes of death in the USA in the same year: heart disease and cancer [28]. Considerable research has shown that modifiable lifestyle factors such as diet, exercise, and refraining from smoking and being overweight can exert a massive reduction, but most people ignore this advice. The US Nurses' Study exemplified how big the beneficial effect of healthy living can be [29]. The greater the number of low-risk lifestyle factors women had, the lower their risk of heart attack or stroke was. The implications are that, in women, 82% (95% confidence interval 58 to 93%) of heart attacks and 74% (95% confidence interval 55 to 86%) of heart attacks or strokes are preventable by having a good lifestyle. Despite widespread advice about healthy living, four out of five US citizens have lifestyles that put them at increased risk of heart attack and stroke [30].

When the number of deaths from heart disease (684,000 in the USA in 2003) and stroke (158,000) is so large, the implication is that people in general are content with large numbers of avoidable deaths from some causes, which are well known, largely within their control, and perhaps 'natural'. Yet the same people can cavil over extremely remote risks from nuclear power plants, electricity power lines or mobile phones, over which they have, or believe they have, no control, and which are man-made. New risks need to be put into perspective, and this might be considered an important aspect of evidence-based decision-making that has, as yet, received little attention.

The lesson is that, in practice, patients' response to risk is influenced by more than just hard facts. It may be that if risks were presented in an appropriate context, people's attitudes to risk or behaviour might change.

#### Antecedents and consequences

How individuals assess and process risk information is dependent on their circumstances or medical condition at that time. Attitudes and choices about an intervention depend on the state of illness as well as on the perceived benefits that accompany the risk. For instance, adherence to statins or low-dose aspirin for cardioprotection is low. In the USA it is estimated that only about 50% (1 in 2) of patients continue at 6 months, and 30 to 40% (1 in 3) at 1 year [31], and in the UK 50% (1 in 2) of patients prescribed low-dose aspirin have discontinued within a year [32]. This low adherence may be a combination of low expectation of personal benefit for therapies that are measures of prevention, combined with an adverse event that crosses a consequential boundary for the individual.

Where benefit is greater and more tangible, adherence is likely to be higher, even if adverse events are common. Thus in renal transplant patients, only 15% (1 in 7) were non-adherent to immunosuppressants under stringent criteria [33]. The consequence of non-adherence, rejection of a transplanted kidney, was particularly significant, with an absolute risk increase averaging 26% (1 in 4) over a number of studies.

At face value, the idea of placing a catheter in the epidural space alongside the spinal cord does not seem to be a good one, because of the possibility of direct physical damage, indirect physical damage from a haematoma, or infection, any of which could result in transient or permanent neurological damage. Yet 2.4 million of the 4 million births in the USA every year involve epidural analgesia, a procedure accepted because the benefits of pain relief are immediate and great, the risk is small (persistent neurological injury 1 in 240,000; transient 1 in 6,700 [34]), and not all risks are directly connected with the epidural. Childbirth is common, women may have experienced an epidural themselves or be familiar with the experience of others, and all these antecedents influence the acceptance of a low risk.

Perhaps one of the most striking examples of antecedent effects on risk behaviour is smoking cessation. In primary care, nurse interventions for smoking cessation had no effect, with about 4% (1 in 25) quitting with or without intervention by a nurse. In hospital settings and patients after cardiac surgery, heart attack, or with cancer there were high quit rates (25%; 1 in 4) without intervention by a nurse, and even higher rates (32%; 1 in 3) with an intervention [35]. The difference between the presence and the absence of serious illness changed attitudes of smokers towards quitting and therefore changed the effects of intervention to help stop smoking. Attitudes to risk and measures of prevention seem to change when an event becomes a more immediate problem.

### Presenting risk

To find studies of any description regarding risk perception and presentation, a number of broad, free-text searches were undertaken with PubMed (up to September 2006). Combinations of words, for instance 'risk AND presentation', or 'risk AND communication' were used, and any original studies or reviews likely to be pertinent were obtained, in as much as they related to communicating medical risks. Bibliographies were examined to uncover other relevant studies, because electronic searching alone is inadequate [34,36].

Studies found were used to inform thinking about risk and risk communication, rather than to constitute a formal systematic review. The wide range of issues relating to risk perception and presentation, and the fragmented and often sparse research literature, rules out a conventional systematic review.

#### Frequency, probability, and words

Probability, in terms of simple frequencies or odds, is often used to describe or communicate risk, sometimes in numbers, often with associated verbal descriptors (common, rare, negligible), and sometimes also with graphical presentations. Some of the more commonly used risk scales have been reviewed by Adams and Smith [37]. There is an assumption, perhaps unstated, that we can couple the numbers and words externally so that their relationship remains fixed.

Patients are known to respond differently to how adverse events are presented. For instance, the patients estimated the likelihood of an adverse event as three to nine times greater with verbal rather than numerical information [38]. Similar differences can be seen in professionals. Graduate students and healthcare professionals in Singapore were asked to match frequency with one of six phrases, from very common to very rare, when a hypothetical situation about adverse events of an influenza vaccine was presented to them in either a probability format (5%) or a frequency format (1 in 20) [39]. With either format of numerical presentation, a risk of 1 in 20 was described verbally from rare to very common, with somewhat more consistency for frequency format than probability.

The European Union has guideline descriptors for the frequency of an adverse event, with verbal descriptors linked to frequency. Thus very common is more than 10% (or greater than 1 in 10) and very rare is less than 0.01% (less than 1 in 10,000). Four studies involving more than 750 people demonstrate that people invariably grossly overestimate frequency from these verbal descriptors [40]. Overestimation occurred at all frequencies, but for the very rare adverse events they were overestimated by at least 400-fold.

The way in which we perceive and process numbers seems to be very different from how we perceive and process words, and different in different people. Moreover, different numbers are linked to similar words in different scales; for instance, the European Union descriptors are not the same as those proposed by Calman [41] or others (Table 1).

**Table 1:** Risk frequency and various verbal descriptors

Frequency range (1 in)	EU descriptors	Calman verbal scale	Calman descriptive scale	Paling perspective scale
1–9	Very common			Very high
10–99	Common	High	Frequent, significant	High
100–999	Uncommon	Moderate		Moderate
1,000–9,999	Rare	Low	Tolerable, reasonable	Low
10,000–99,999	Very rare	Very low		Very low
100,000–999,999		Minimal	Acceptable	Minimal
1,000,000–9,999,999		Negligible	Insignificant, safe	Negligible

Data are taken from [41] and other sources. EU, European Union.

#### Framing risk for patients

When patients are provided with information about drug therapy or surgery, the way in which information is provided can affect patient decisions in a major way, and the extensive literature has been reviewed, especially in terms of benefits or losses, situation, and context [42]. Our knowledge of the extent of framing effects on patients and outcomes is limited by small numbers of relatively small studies [43].

Patients respond very differently depending on how data about benefits of therapy are framed. Hypertensive patients only rarely would have refused hypertensive therapy when information about efficacy was presented as relative risk reduction, but refusal rose to 23% (1 in 4) for absolute risk reduction, 32% (1 in 3) for number needed to treat, and 56% (6 in 10) with information presented as patient-specific probability of benefit [44]. The choice between having surgery or a cast for a fracture [45], or different types of surgery [46], is influenced by framing effects of different types of data presentation, verbal renderings of outputs such as relative risk reduction, or number needed to treat.

It is not only patients who respond differently to data depending on presentation or framing. A number of studies have documented the fact that relative presentation (like relative risk reduction) has a much greater influence on professionals' decision-making than absolute risk difference or number needed to treat. This is true for purchasers [47], hospital doctors [48], general practitioners [49,50] and pharmacists [51]. Although a systematic review of randomised trials supports this general finding, it also indicates that framing is susceptible to modification by other factors [52].

#### Pictorial representation of risk

Calman and Royston [53] reviewed a number of different ways of explaining risk, including pictorial representations involving logarithmic scales, expressing results in terms of distance, or population, and the use of visual presentation. Paling [54] had already suggested a visual presentation of risk with logarithmic scales, and later expanded risk presentation with a number of different presentations into the clinical, rather than the predominantly environmental, field [55,56]. Other types of representation have been suggested, based, for instance, on number needed to treat [57], although women favoured simple bar charts for the presentation of absolute lifetime risks [58].

Other suggestions have expanded use of the scales, with some contextualising information [59], into mainly anaesthetic [37] or obstetric and gynaecological risks [60]. The utility of logarithmic scales such as the Paling scale in delivering better information about risk has been tested at least once [61]: both visual and comprehensive written information on transfusion risks improved patient knowledge to the same extent. This agrees with a systematic review, which also showed that decision aids improved patient involvement, knowledge, and realistic expectation of benefits and harms [62].

**Figure 2:**
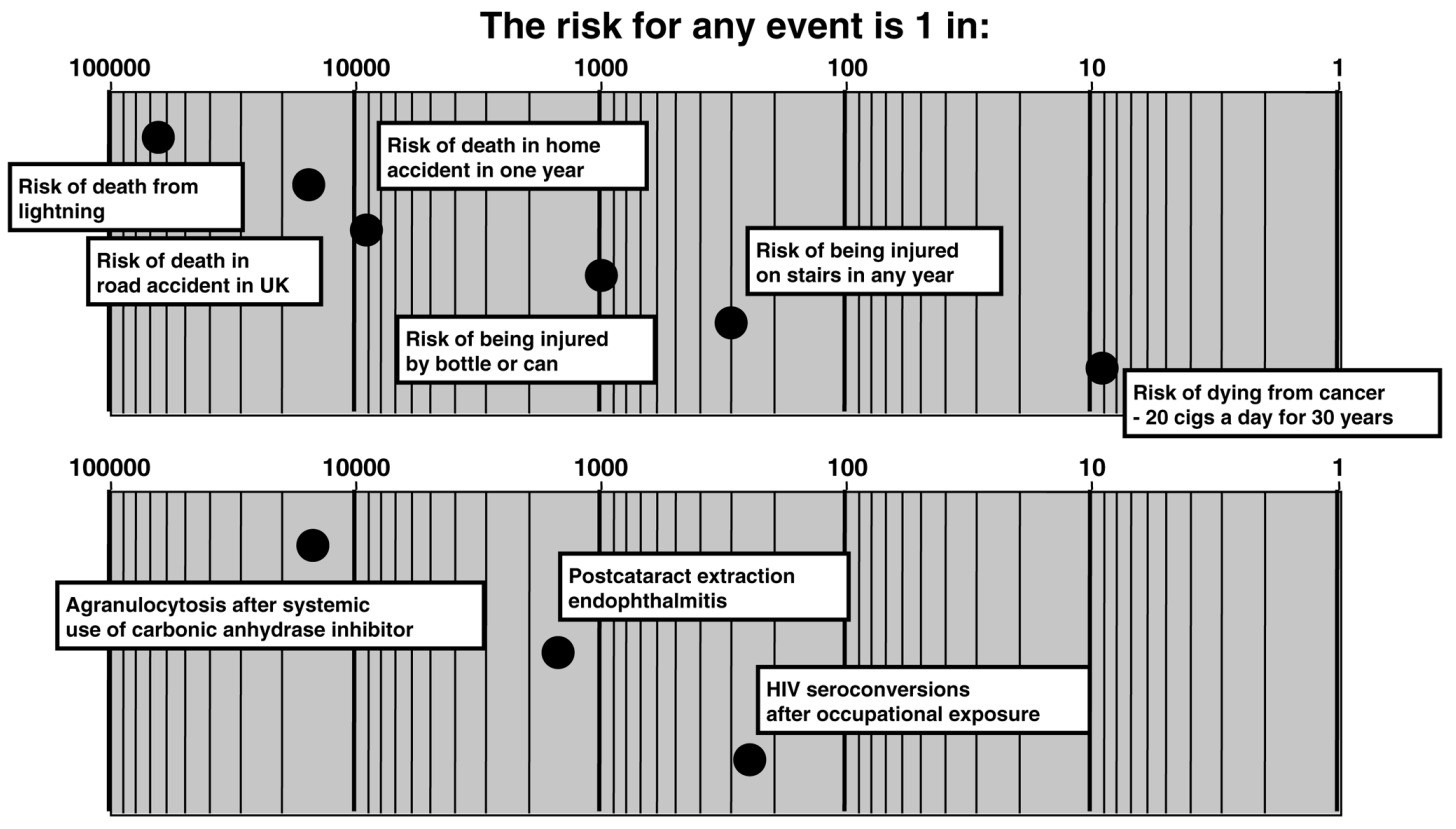
Early attempt to contextualise risk [63]. Cigs, cigarettes.

Visual risk scales have not been used extensively. Scales might be made more relevant by adding contextualising information to medical risk (Figure 2) [63]; contextualising anchors were chosen only because they seemed useful at the time, and they can be criticised for not necessarily being relevant to the specific risks arising from the intervention. Although the risks may be contextualised, the wrong context was used.

It is difficult to obtain good information for all grades of risk or adverse event, with their various dimensions. Population data are available, though, on death from various causes. Serious but rare adverse events are often associated with death. Myocardial infarction, gastrointestinal bleeding, and rhabdomyolysis, for example, can be fatal or non-fatal, and the fatality rate is known. It is therefore possible to link the risk of death associated with an intervention to other, common risks that we face as individuals.

A series of examples follow, using a vertical form of the Paling Perspective Scale, populated with numerical and verbal descriptors of risk, together with information on the risk of death from various causes taken from US data in 2002 [27,28]. The contextualising examples include high mortality risk from heart disease (about 1 in 400 per year for US adults, although obviously skewed to older people), and death from any accident (about 1 in 2,000). Low risks include death from an automobile accident (about 1 in 20,000) or from any fall (about 1 in 70,000). Very low risks include death from firearm (about 1 in 300,000) or in a cataclysmic storm or lightning (about 1 in 3,000,000).

**Figure 3:**
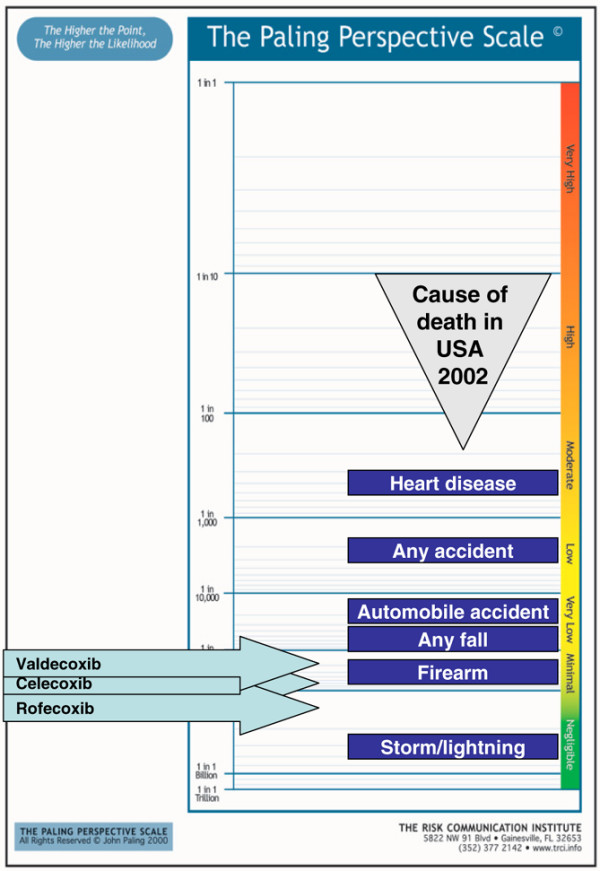
Risk of serious skin reactions with coxibs [64].

**Figure 4:**
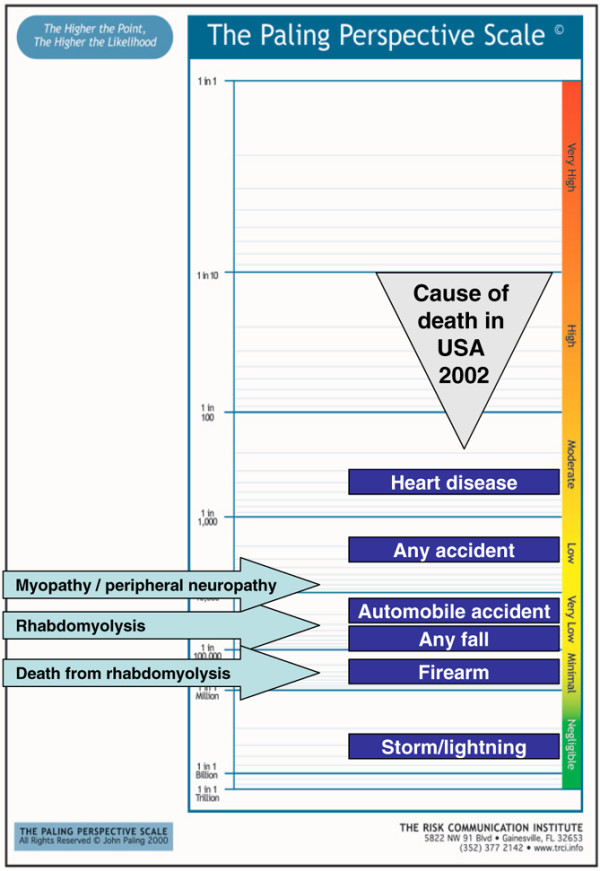
Risk of myopathy, rhabdomyolysis and death from rhabdomyolysis with statins [65].

**Figure 5:**
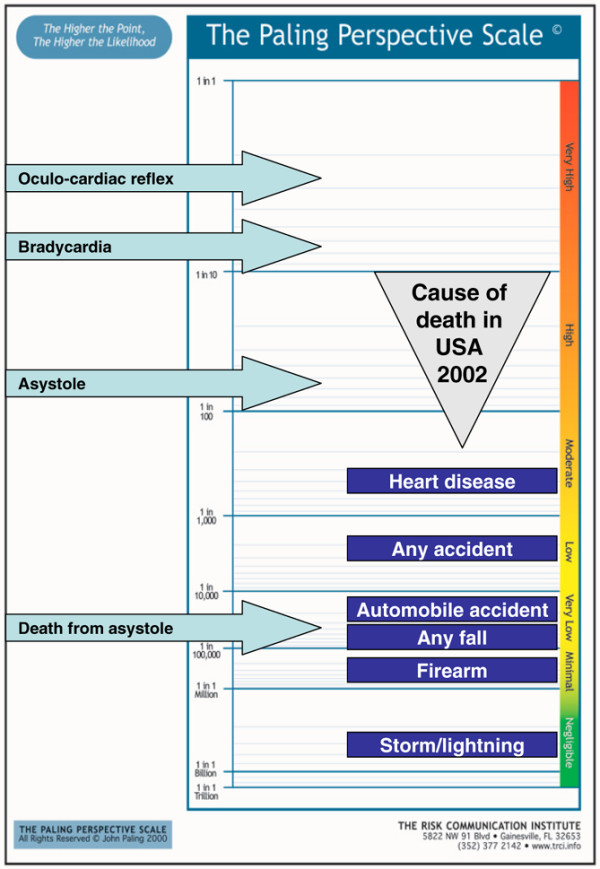
Risk of cardiac adverse events, including death, associated with use of propofol anaesthesia [66].

**Figure 6:**
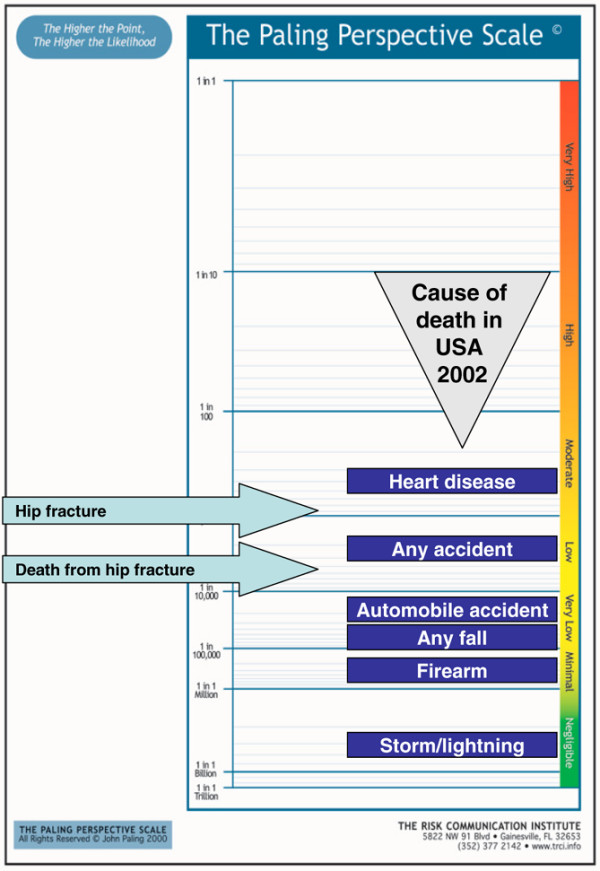
Risk of hip fracture associated with proton pump inhibitor [67]. Use for 1 year or more in people aged over 65 years.

**Figure 7:**
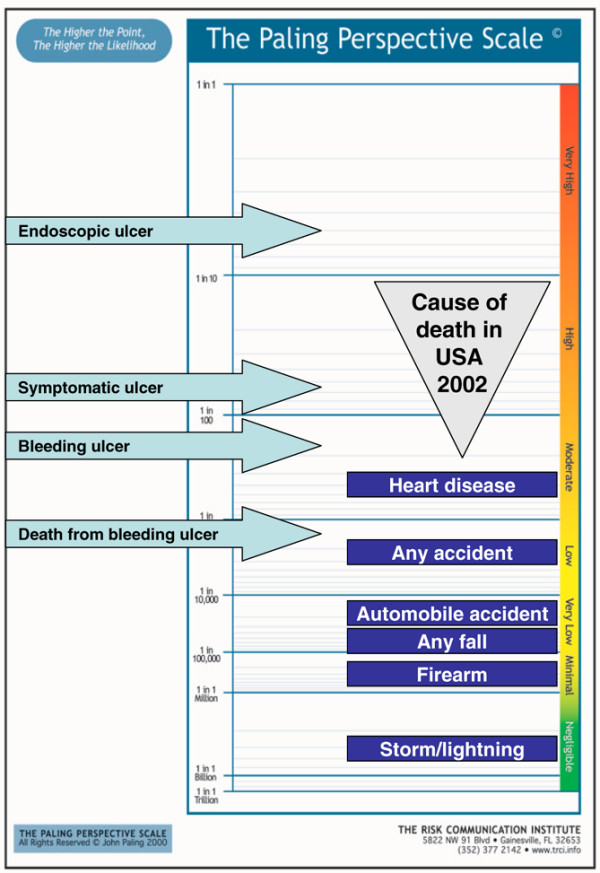
Risk of death from gastrointestinal bleeding with NSAID or full-dose aspirin [68]. Use for 2 months or longer.

Data on risk of mortality from medical interventions were taken from systematic reviews or large observational studies, and, if needed, mortality was calculated from the rate of the adverse events and the known or estimated mortality rate from that event. The examples are as follows:

1. Risk of serious skin reactions with coxibs [64]. Because these data come from adverse event reporting they almost certainly underestimate the true risk, but from these data the risks varied between 1 in 300,000 for valdecoxib, to 1 in 1,000,000 for celecoxib, and 1 in 1,700,000 for rofecoxib (Figure 3).

2. Risk of muscle adverse events of statins, including rhabdomyolysis and death from rhabdomyolysis [65]. The risk of death from rhabdomyolysis is about 1 in 300,000 a year (Figure 4).

3. Risk of cardiac adverse events, including death, associated with use of propofol anaesthesia [66]. Here the risk of death from asystole was estimated at about 1 in 70,000 (Figure 5).

4. Risk of hip fracture associated with use of proton pump inhibitor for 1 year or more in people aged over 65 years. Data from the UK General Practice Database suggesting a doubling of risk [67] are supported by evidence of an increased risk seen in Denmark [68]. The risk of death from hip fracture while using a proton pump inhibitor is 1 in 4,500 (Figure 6).

5. Risk of death from gastrointestinal bleeding with NSAID or full-dose aspirin for 2 months or longer [69]. This gave a risk of death of 1 in 1,200 (Figure 7).

The presentation of risk with these methods – a common outcome of death, and the Paling Perspective Scale – requires that a body of evidence is available to allow the appropriate calculations. As the rather disparate examples in Figures 3 to 7 show, it is unusual to have a coherent set of data available for a single topic because the amount or extent of evidence is not available. A notable exception is the case of NSAIDs and coxibs, and the outcomes of gastrointestinal and cardiovascular events, which have been the subject of extensive investigation in both randomised trials and a retrospective meta-analysis of them, and meta-analyses of substantial numbers of observation studies examining the use of NSAIDs and coxibs in the community.

### Death from gastrointestinal and cardiovascular events with NSAIDs and coxibs

Systematic reviews and meta-analyses of observational studies published since 2000 reporting either upper gastrointestinal bleeding or cardiovascular events with particular NSAIDs and/or coxibs were used for relative risk estimates. For upper gastrointestinal bleeding, we also used individual observational studies published since 2000, because searching uncovered only a single systematic review [6], which was devoid of information on coxibs.

The search strategy avoided meta-analyses of randomised trials, because many of the data in those came from trials with higher than licensed doses of coxibs, and maximum daily doses of NSAIDs. This does not reflect clinical practice, in which guidance is to use the lowest dose possible for the shortest possible time. By contrast, observational studies reflect actual clinical practice, including dose, more accurately, and also have the benefit of being larger, with many more events.

We also sought studies that would provide information on background rates of upper gastrointestinal bleeding or cardiovascular events in the absence of use of NSAIDs or coxibs, initially from studies in the original search, but supplemented with additional searches and the use of bibliographies. In addition, we required information on the likely mortality rate for upper gastrointestinal bleeding and cardiovascular events to provide a suitable and consistent context. The background rate of events, the relative risk with NSAID or coxib, and the probability of dying could then be used to calculate the additional risk of death from gastrointestinal and cardiovascular events associated with the use of particular NSAIDs and coxibs.

#### Data on event rates for individual NSAIDs and coxibs

Table 2 summarises the main findings. One systematic review and meta-analysis of upper gastrointestinal bleeding [6] collected information from observational studies of NSAIDs in the 1990s but was devoid of coxib data. Data on coxibs and additional NSAIDs were available in four individual studies published subsequently [5,7,70,71]. Estimates of relative risk were generally in good agreement. The influence of duration of use was uncertain; one individual study found higher risk with short-term versus long-term use [5], although no relationship between increased event rate and duration was evident in a systematic review [6].

**Table 2:** Relative risk (95% confidence interval) for serious upper gastrointestinal bleed or myocardial infarction

Information source	Relative risk compared with non-use of coxib or NSAID					
	
	Ibuprofen	Naproxen	Diclofenac	All NSAIDs	Celecoxib	Rofecoxib
Upper GI bleed [6]	1.9 (1.6–2.2)	4.0 (3.5–4.6)	3.3 (2.8–3.9)	4.2 (3.9–4.6)		
Upper GI bleed [5]	1.7 (1.1–2.5)	9.1 (6.0–14)	4.9 (3.3–7.1)			
Hospital admission [7]				4.0 (2.3–6.9)	1.0 (0.7–1.6)	1.9 (1.3–2.8)
Upper GI bleed [70]				3.3 (2.4–4.4)	1.3 (0.7–2.8)	2.1 (1.2–3.5)
Upper GI bleed [71]	4.1 (3.1–5.3)	7.3 (4.7–11.4)	3.1 (2.3–4.2)	5.3 (4.5–6.2)	1.0 (0.4–2.1)	2.1 (1.1–4.0)
CV events [4]	1.07 (1.02–1.12)	0.98 (0.92–1.05)	1.44 (1.32–1.56)	1.09 (1.06–1.13)	0.96 (0.90–1.02)	1.26 (1.17–1.36)
CV events [8]	1.07 (0.97–1.18)	0.97 (0.87–1.07)	1.40 (1.16–1.70)	1.10 (1.00–1.21)	1.06 (0.91–1.23)	1.35 (1.15–1.59)

Results for NSAIDs and coxibs were compared with non-use, from observational studies. These did not, or were unable to, produce dose-specific results. Bold lines represent relative risks or equivalent from systematic reviews and meta-analyses. Coxib, cyclooxygenase-2 inhibitor; NSAID, non-steroidal anti-inflammatory drug; GI = gastrointestinal; CV = cardiovascular.

Two systematic reviews provided essentially identical estimates of relative risk for cardiovascular events [4,8] (Table 2). One further systematic review [72] was without pooled estimates for individual drugs.

We used figures for relative risk of upper gastrointestinal bleeding from the meta-analysis for NSAIDs, and an average figure from observational studies for coxibs. We used relative risks for cardiovascular events from the meta-analysis with the largest body of data [4]. Results of both systematic reviews were broadly in line with a pooled analysis of cardiovascular events in randomised trials [3], namely a significant difference between coxibs and placebo in trials of colorectal polyps (but not dementia or arthritis trials, in which background event rates are higher), and an increase with doses of rofecoxib above 25 mg a day.

#### Background rates of events without NSAID or coxib

The main patient-specific influences on the background incidence of both gastrointestinal bleeding and myocardial infarction are age and sex.

For serious upper gastrointestinal bleeding or perforation in non-users of NSAIDs, a systematic review of epidemiological studies [73] suggests a rate of 1 in 1,000 persons a year, although at age 60 years a higher rate of about 2 or 3 per 1,000 would apply, similar to that of a large survey in Spain [71]. A cohort study in Canada [7] showed matched non-users (mean age 75 years) to have a rate of 2.2 per 1,000.

As regards non-users of NSAIDs, Mamdani and colleagues [74] reported a rate of myocardial infarction of 8.2 per 1,000 person years. This is in line with reports of the incidence of acute myocardial infarction without including pre-admission deaths from Holland [75] and England [76].

We used background rates of 2.2 per 1,000 for gastrointestinal bleed and 8.2 per 1,000 for myocardial infarction as being typical of non-users of NSAIDs or coxibs selected as controls in large observational studies.

#### Mortality from upper gastrointestinal bleeding and cardiovascular events

Gastrointestinal bleeding carries a risk of death of about 6% according to a large, recent, Spanish observational study with most patients aged over 60 years [77], up to 14% in a recent Dutch study [78], and in the range of 6 to 12% in a meta-analysis combining randomised trials and observational studies [69].

About 1 in 3 people who have a heart attack die before they reach hospital [79,80]. Mortality within 30 days of a hospital admission with myocardial infarction was 11% in a recent Danish study of people aged 30 to 74 years [81]. However, sudden cardiac death rate before hospital admission is higher than this, with overall 28-day mortality, including sudden cardiac death outside hospital, of about 40% [76]. In Finland the 28-day case mortality rate for men was 34% and for women it was 20% [82].

To estimate mortality for risk calculations we chose to use rounded estimates of 10% mortality for gastrointestinal bleeding and 30% for myocardial infarction.

#### Calculating competing risks

Table 3 shows calculations of risk for individual NSAIDs and coxibs compared with non-use, using the background rates of 2.2 per 1,000 for gastrointestinal bleed and 8.2 per 1,000 for myocardial infarction [4,15]. It provides an indication of the likely risks for an average patient. The calculations were for additional number of events, the likely number of additional deaths, and the frequency of those deaths.

**Table 3:** Additional gastrointestinal bleeding events and myocardial infarction associated with using NSAIDs and coxibs

Event and drug	Relative risk	Additional events per 1,000	Additional deaths per 1,000	Frequency (1 in)
Gastrointestinal bleeding (background rate 2.2 per 1,000)				
Ibuprofen	1.9	1.98	0.20	5,051
Naproxen	4.0	6.60	0.66	1,515
Diclofenac	3.3	5.06	0.51	1,976
All NSAIDs	4.2	7.04	0.70	1,420
Celecoxib	1.1	0.22	0.02	45,455
Rofecoxib	2.0	2.20	0.22	4,545
Myocardial infarction (background rate 8.2 per 1,000)				
Ibuprofen	1.07	0.57	0.17	5,807
Naproxen	0.98	-0.16	-0.05	-20,325
Diclofenac	1.44	3.61	1.08	924
All NSAIDs	1.09	0.74	0.22	4,517
Celecoxib	0.96	-0.33	-0.10	-10,163
Rofecoxib	1.26	2.13	0.64	1,563

Any dose of drug was allowed in the data, and the table additionally shows the rate and frequency of additional events. The calculations used a mortality rate of 10% for gastrointestinal bleeding and 30% for cardiovascular events. NSAID, non-steroidal anti-inflammatory drug; coxib, cyclooxygenase-2 inhibitor.

For example, for gastrointestinal bleeding with a background rate of 2.2 bleeds per 1,000 patients per year, use of ibuprofen would result in 1.98 extra bleeds (calculated as (2.2 × 1.9) -2.2, or 4.18 -2.2, or 1.98). With a death rate of 10%, this would mean 0.2 additional deaths per 1,000 per year, at a frequency of 1 in 5,051 (calculated as 1,000 ÷ (1.98 ÷ 10)). Results for other drugs or outcomes were derived similarly. Where there was no significant difference between use of NSAID or coxib and non-use, a risk frequency of 1 in 100,000 was assumed.

#### Presenting contextualised risks

**Figure 8:**
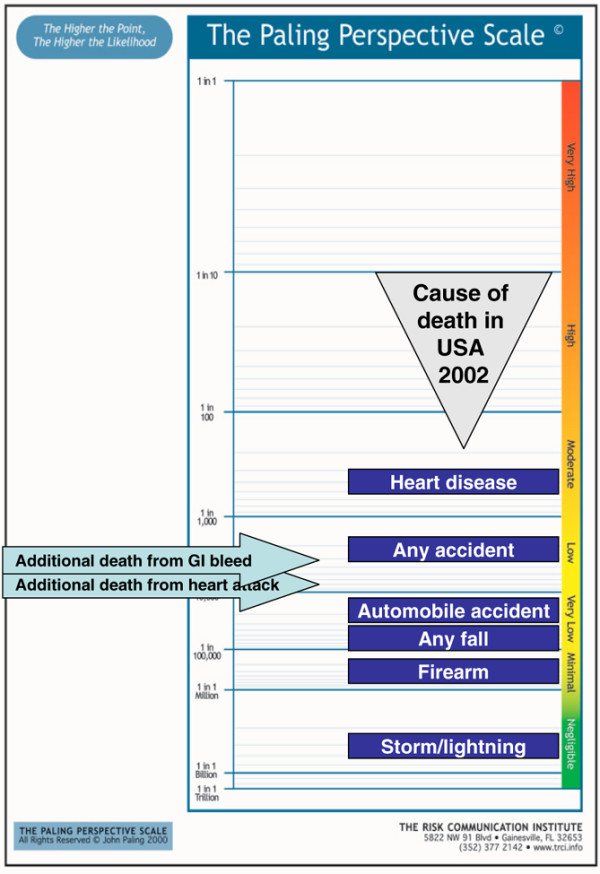
Additional risk of dying from an upper gastrointestinal bleed or cardiovascular event with ibuprofen. GI, gastrointestinal.

**Figure 9:**
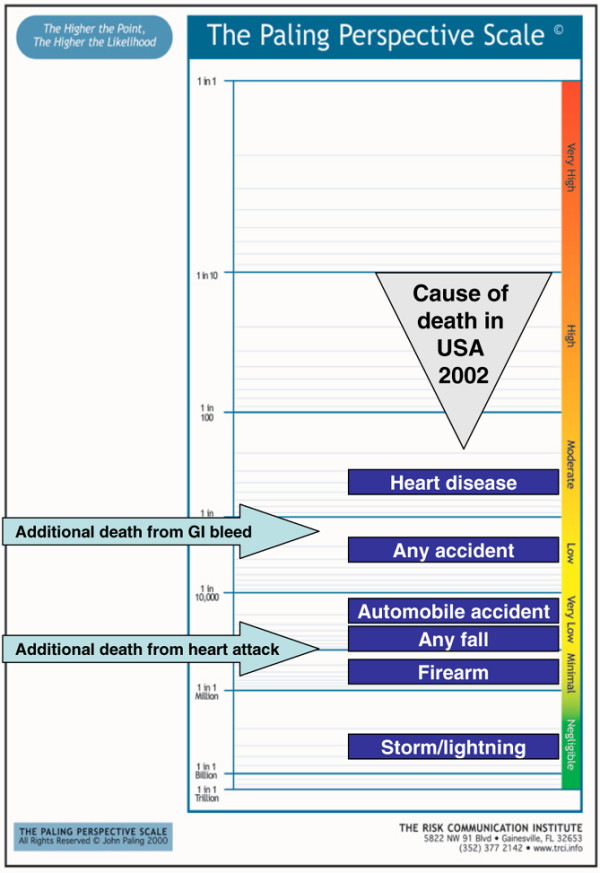
Additional risk of dying from an upper gastrointestinal bleed or cardiovascular event with naproxen. For representational purposes an additional risk of 1 in about 100,000 was assumed where there was no numerically increased cardiovascular risk. GI, gastrointestinal.

**Figure 10:**
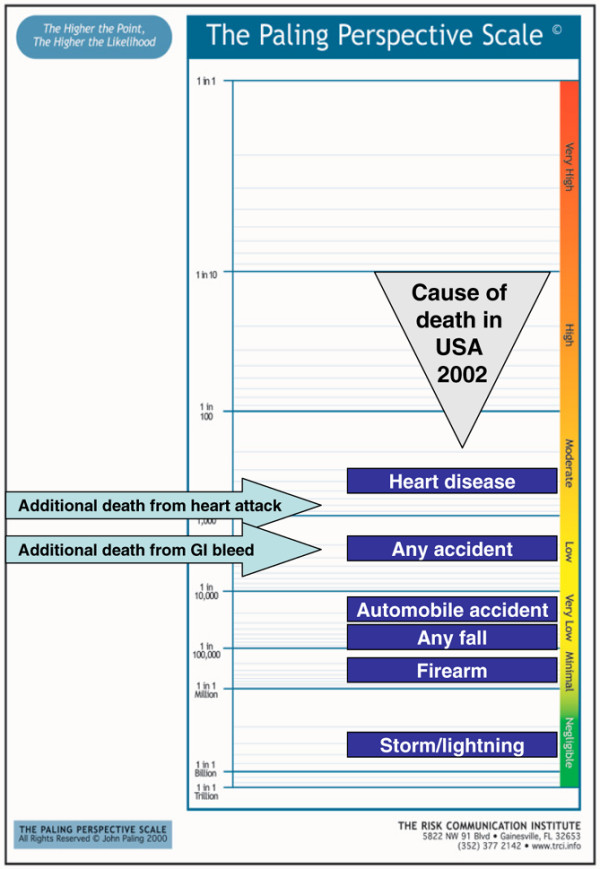
Additional risk of dying from an upper gastrointestinal bleed or cardiovascular event with diclofenac. GI, gastrointestinal.

**Figure 11:**
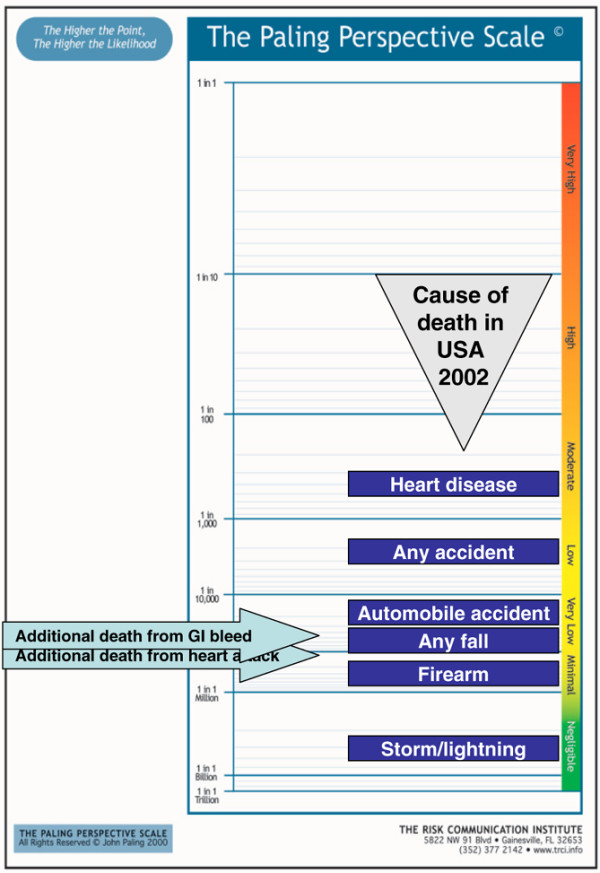
Additional risk of dying from an upper gastrointestinal bleed or cardiovascular event with celecoxib. For representational purposes an additional risk of 1 in about 100,000 was assumed where there was no numerically increased risk, here for either risk. GI, gastrointestinal. GI, gastrointestinal.

**Figure 12:**
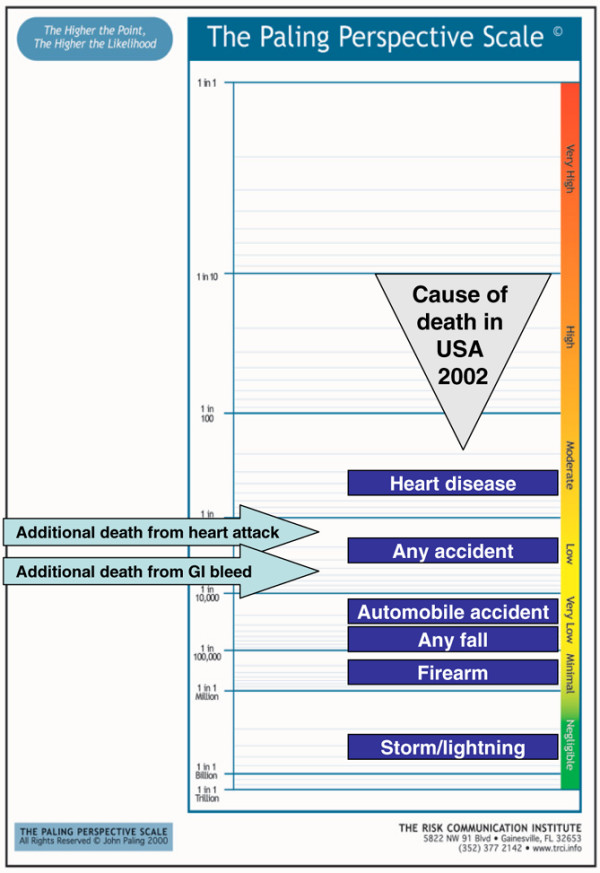
Additional risk of dying from an upper gastrointestinal bleed or cardiovascular event with rofecoxib. GI, gastrointestinal.

Figures 8 to 10 show the additional risk over background of dying with an upper gastrointestinal bleed or cardiovascular event for users of ibuprofen, naproxen and diclofenac, respectively. Figures 11 and 12 show the same information calculated for celecoxib and rofecoxib. In these representations, the events have been described as gastrointestinal bleeding or heart attack, for simplicity, and to be less technical to facilitate possible use with patients rather than professionals.

The figures show that additional risks can vary from moderate (gastrointestinal bleeding with naproxen and cardiovascular risk with diclofenac) through to very low or negligible (gastrointestinal bleeding with celecoxib and cardiovascular risk with naproxen and celecoxib). There are considerable differences between the five drugs.

**Figure 13:**
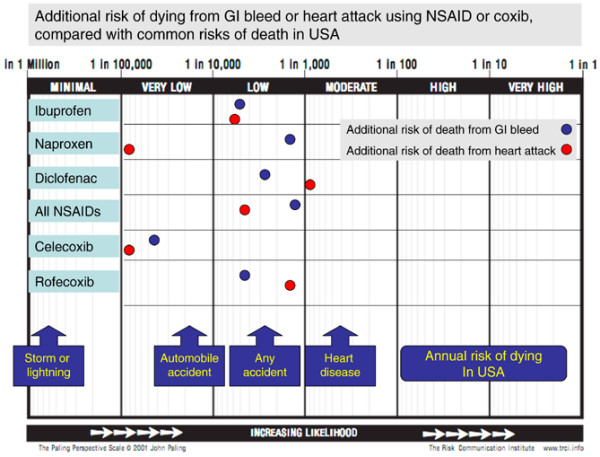
An alternative version of the Paling Perspective Scale. It puts the five drugs from Figures 8 to 12 together with all NSAIDs combined, on a single, horizontal, version. GI, gastrointestinal; NSAID, non-steroidal anti-inflammatory drug; coxib, cyclooxygenase-2 inhibitor.

An alternative version of the scale (Figure 13) presents the five drugs, together with all NSAIDs combined, on a single, horizontal, version. This might allow easier comparison, both between drugs and with some acceptable level of risk provided by contextualising information.

## Discussion

It is important to recognise that the method of presenting risk outlined in this paper is only one way in which the relative consequences of treatment might be shown. Whether the method is useful to patients or professionals in some of the contexts shown is not known, and we stress that it still has to be evaluated. The value of the method is also dependent on the quality and quantity of evidence about risk in any given situation.

That said, patient-led healthcare means that patients need to be supported in making choices about, and taking control of, their health and healthcare. Not only must services evolve to provide personalised care by listening and responding to patients, but information also needs to be provided to them to help in decision-making. Patients react adversely to hypothetical risk [83], and providing information about a rare but serious risk of treatment may lead them to make different judgements. When asked, patients want to know about even rare risks of adverse events [17].

Most adverse events are mild, reversible and predictable, although common. They pose some prospect of discomfort and may lead to drug discontinuation if they cannot be tolerated, but they are not dangerous. More problematic are those adverse events that are serious, irreversible and unpredictable. They will be rare because no drug could be marketed if these adverse events were also common. It is these rare but serious events that attract attention. Paradoxically, more effective and widely used medicines are more likely to attract pressure for bans based on adverse events [84]. This is because with only a few hundred or a few thousand people using a drug, a rare but serious adverse event at the 1 in 100,000 level would never attract attention. By contrast, use in 2 million people would result in 20 events that could well attract attention.

Where a medical intervention is performed for major life-saving or life-enhancing purposes (such as cardiac revascularisation or joint replacement), possible adverse events are offset by significant, important and largely immediate benefit; only major adverse events, such as mortality, are likely to form a part – and perhaps only a small part – of decision-making. By contrast, where an intervention delivers less immediate benefits or where there are alternative therapies available, the risk of avoidable adverse events becomes a more significant part of decision-making.

Herein lies the problem. Rare risks of major, irreversible, consequences are by their nature difficult to measure precisely. To this uncertainty must be added the uncertainty of how information on the risk can be presented in a way that is understood. This is especially true when there is a background rate in the population, which we must know or guess, and we then have to apply an imprecise relative risk, to make judgements about the severity of different outcomes. It all makes for complex mental arithmetic, and a representation of the additional risk faced compared with other risks we assume in life has obvious benefits, especially when the event is common to all.

Figures 3 to 7 present a series of risks of death associated with treatments in the range (roughly) of 1 in 1,000,000 to 1 in 1,000, all of which would be regarded are rare. The information about life events regarded as rare allows that to be interpreted and judged, and to provide a context in which individual decisions can be made. Any judgement will depend not just on the level of risk but also on the benefits. The fact that a rare death from a cutaneous adverse event from valdecoxib (Figure 3) is judged differently from a similar risk of death from rhabdomyolysis from a statin (Figure 4) is not necessarily inconsistent.

There are few easy cases when it is possible to say that a proposed therapy is universally effective or safe, and especially both effective and safe. Most situations are complex, and none apparently more so than that of choice of NSAID or coxib for chronic pain. The examples here have considered only additional risk of death from gastrointestinal bleeding or cardiovascular events, compared with different background rates without drug therapy. Other levels of risk could have been chosen, including non-fatal outcomes. Moreover, we have deliberately ignored renovascular events, congestive heart failure, lower bowel problems, anaemia, hypertension and other adverse events, more and less severe, that might have been included, especially from individual patient meta-analysis of randomised trials [85].

In any therapeutic area there are competing risks and benefits of alternative therapeutic interventions. This paper explores ways in which risk of just two possible adverse events can be displayed for several NSAIDs and coxibs that display numerically quite different risks from each other. We have no evidence about how best to convey these to patients in a way that will be fully comprehended, nor have we the evidence to personalise risk presentation for the individual, so we have to rely on average results. This is an important omission, because consequences of treatment are likely to differ for individual patients, and this now has theoretical underpinning with regard to coxibs [86].

There are other ways in which risk may be presented. A large observational study of more than 500,000 over-65s in Canada [87] examined both myocardial infarction and gastrointestinal bleeding to produce a combined estimate of risk. It used a different method, but for individual drugs and for patients taking or not taking low-dose aspirin. Alternatively, data from meta-analyses of randomised trials have been used to present annualised risk estimates for placebo, pooled NSAIDs, and coxibs [88].

Attitudes of individuals presented with information about possible risks and benefits of treatments will differ as they see the possible consequences for themselves differently. The product of information presented, their own experience, and emotional factors results in widely differing choices between individuals [89,90]. All communicated facts will finally be filtered by emotions before a decision is made. We have to acknowledge that there is a danger of focusing more on how to calculate and present numerical conclusions about risk, while ignoring our ignorance of other aspects of decision-making.

There are a number of possible next steps. Contextualised risk presentations such as these need to be refined. It may well be that other forms of presentation, or different contextualising risks, would make them clearer and more relevant for professionals and patients. Risk presentation methods are likely to have different degrees of success with people of different backgrounds, languages and cultures. We might consider whether it is possible to develop risk presentations with greater utility for physicians. An example is the Joint British Societies coronary risk prediction charts found in every copy of the British National Formulary.

## Conclusion

We suggest one way of communicating information about the risk of rare adverse events that can result in death, by combining words, numbers, pictures and context. The area of risk communication requires significantly more research because the communication of risk has a limited knowledge base, irrespective of whether it is common or rare, serious or inconsequential.

## Abbreviations

Coxibs = selective cyclooxygenase-2 inhibitors; NSAIDs = non-steroidal anti-inflammatory drugs.

## Competing interests

RAM and HJM have received research grants, consulting, or lecture fees from pharmaceutical companies, including Pfizer, MSD, GSK, AstraZeneca, Grunenthal, Menarini and Futura. The authors have also received research support from charities and government sources at various times. RAM is the guarantor. JP earns his living from teaching about and consulting on risk communication with doctors, healthcare organisations and pharmaceutical companies. As part of his services, JP offers special visual aid formats for licensing by commercial organisations. However, free web-based programs enable unrestricted access to all parties to build and print customised versions of JP's main decision aids for non-commercial purposes such as all individual doctor-patient communications and for trial and evaluation (see ). No author has any direct stock holding in any pharmaceutical company.

## Authors' contributions

RAM was involved with the original concept, planning the study, searching, analysis and preparing a manuscript. SD and HJM were involved with planning, data extraction, and writing. JP was involved with planning, writing and visual aids formats to explain risks. All authors read and approved the final manuscript.
